# Optimized extraction and quality evaluation of Niger seed oil via microwave‐pulsed electric field pretreatments

**DOI:** 10.1002/fsn3.1396

**Published:** 2020-01-28

**Authors:** Nazanin Maryam. Mohseni, Habibollah Mirzaei, Masoumeh Moghimi

**Affiliations:** ^1^ Department of Food Science and Technology Sari Branch Islamic Azad University Sari Iran; ^2^ Department of Food Materials and Process Design Engineering University of Agricultural Sciences and Natural Resources Gorgan Iran; ^3^ Department of Chemistry Gonbad Kavoos Branch Islamic Azad University Gonbad Kavoos Iran

**Keywords:** microwave, Niger seeds, oil extraction, pulsed electric fields, tocopherols

## Abstract

In this study, oil extraction from Niger seeds was evaluated with different microwave irradiation times (0–200 s) and pulsed electric fields (PEF) intensities (0–5 kV/cm) as pretreatments. Then, oil extraction was completed with a screw press at different rotation speeds (11–57 rpm). Quality parameters including extraction efficiency, acidity and peroxide values (PVs), chlorophyll, and phenolic contents along with fatty acid profiles and tocopherol levels of the extracted oils were determined as responses. With enhancements in microwave time, PEF intensity and press rotation, the chlorophyll contents, acidity/PVs, and total phenolics of oils increased similar to oil extraction efficiency although it was reduced later. The optimized conditions selected by response surface methodology were determined as 156.23 s, 1.18 kV/cm, and 20 rpm for microwave time, PEF intensity and press speed, respectively. Fatty acid analysis revealed that linoleic acid was the most predominant fatty acid in the extracted oil. Application of the mentioned pretreatments may lead to a reduction in unsaturated fatty acids and escalation of saturated ones (*p* < .05). High‐performance liquid chromatography results indicated that α‐tocopherols are the most common tocopherols in Niger seed oil and microwave‐PEF pretreatments may lead to 2.79% increase in tocopherols content.

## Graphical abstract

Application of combined pretreatment enhanced the Niger seed oil quality. Alpha tocopherols had the highest quantity among the present tocopherols. Linoleic acid was the predominant fatty acid of Niger seeds oil.

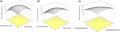

## 1 INTRODUCTION

Oil seeds are important sources of edible, industrial, and medicinal oils which could have bioactive compounds with functional properties for further consideration (Melaku, 2015). Niger seeds with the scientific name of *Guizotia abyssinica Cass* and English name of Niger from Asteraceae family contain high‐quality oils with potential applications in nutraceutical and pharmaceutical fields. It has flowers in yellow color and rarely in light green, fruits in dark color to yellow, hard shell and a white germ (Getinet & Teklewold, 1995). This plant species is cultivated in high scale in Ethiopia and India and in low scale in several countries in Africa, Asia as well as in America. It is also consumed in Europe, United States, Persian Gulf countries including Iran for bird feeding (Melaku, 2015). In different references, 37%–50% oil extraction efficiency for Niger seeds has been reported. The highest unsaturated fatty acids are linoleic and oleic acids, and the most predominant saturated fatty acids are palmitic and stearic acids. Fatty acid profile of this plant is similar to sunflower and safflower with a more quantity of linoleic acid (over than 85%). Niger seeds oil could be used as a replacement for olive and sesame seeds oils in pharmaceutical industries; moreover, it may be used in the production of soaps, paints, grease, and perfumes (Pradhan, Mishra, & Paikary, 1995). Furthermore, this oil contains considerable quantities of antioxidants (Ramadan & Moersel, 2003). Also, the obtained Niger meal through oil extraction process contains about 35% protein and 23% raw fibers (Pradhan et al., 1995), that may be used in feeding of animals and birds as well as humans and also in the preparation of fertilizers (Adarsh, Kumari, & Devi, 2014).

In oil extraction using cold pressing, different parameters including pressure, temperature, or rotational speed of press and moisture content of seeds may affect the extraction efficiency significantly. Another important factor is the appropriate treatment of seeds before extraction in order to produce an oil with the highest quality and efficiency (Azadmard‐Damirchi, Habibi‐Nodeh, Hesari, Nemati, & Achachlouei, 2010). Therefore, novel extraction techniques with the aid of processes such as Pulsed electric fields (PEF) (Zeng, Han, & Zi, 2010), sonication (Jalili, Jafari, Emam‐Djomeh, Malekjani, & Farzaneh, 2018), and microwave (Taghvaei, Jafari, Assadpoor, Nowrouzieh, & Alishah, 2014) have been considered recently.

Microwave irradiations as nonionized electromagnetic waves with the frequency of 300 MHz to 200 GHz result in heating in a selected rout without any heat loss to environment such as heating in enclosed systems. The heating mechanism may lead to a decrease in extraction time compared to other extraction approaches. The effect of this process is performed by two phenomena including ionic transmission and dipole rotation that in most cases happen simultaneously (Jafari, Mahdavee Khazaei, & Assadpour, 2019). In oil seeds, water as a dipolar compound is found in high amounts, along with other compounds such as salt and protein which may act as dielectric compounds (Sultana, Anwar, & Przybylski, 2007). PEF processing includes short‐term pulses (µs) with a high intensity in a strong electrical field on food materials. The application of PEF due to enhancements of cell permeation might increase oil extraction efficiency and there have been some reports on the positive effects of this process on the qualitative and sensory properties of the extracted oil (Bakhshabadi, Mirzaei, Ghodsvali, Jafari, & Ziaiifar, 2018; Boussetta, Soichi, Lanoiselle, & Vorobiev, 2014; Sarkis, Boussetta, Tessaro, Marczak, & Vorobiev, 2015). For instance, PEF has been applied for fat/oil production from microalgae (La et al., 2016). They used PEF with a low energy and expressed that it could replace the previous common fat extraction approaches.

Currently, regarding the population increase and growing demands for healthy and functional oils, it is necessary to work on nonconventional sources of edible oils. On the other hand, novel and emerging processes could have promising results on improving the extraction efficiency and quality of these oils. So, we proposed this study for the first time to optimize the oil extraction from Niger seeds, with the use of microwave‐PEF approach as pretreatments and applying response surface methodology (RSM) as the optimization technique.

## 2 MATERIALS AND METHODS

Niger seeds (containing 40% oil) were provided from Fars province (Iran). Chemical materials including sodium hydroxide, phenolphthalein, Folin–Ciocalteu reagent, methanol, ethanol, hexane, and acetonitrile were purchased from Merck and Sigma companies. Other applied chemicals were of analytical grade.

### 2.1 Pretreatments of Niger seeds and oil extraction

For this purpose, the seeds were firstly treated by microwave with the power of 900 W in different times (0–200 s); then, they were treated by three PEF intensities (0–5 kV/cm). The applied power in PEF chamber was 30 pulse with the width of 20 micro seconds (Kittiphoom & Sutasinee, 2015). Afterward, the oil extraction was completed with a screw press at different rotation rates (11–57 rpm).

### 2.2 Determination of oil extraction efficiency and its quality

Extraction efficiency was determined gravimetrically by the following equation:

(1) $$\text{Oil extraction efficiency}\left(\%\right)\;=\frac{\text{Extracted oil}\left(g\right)}{{\text{Seeds}}^{\prime }\;\text{weight}}\times 100$$

Refractive index of the extracted oils was detected at the temperature of 25°C according to AOCS (Society & Firestone, 1994). Chlorophyll content was measured regarding the method described by Pokorny, Kalinova, and Dysseler (1995). To determine acidity, the method of AOCS 3‐63, 1993 was applied. Firstly, 5 g oil was mixed with 20–30 ml ethanol and titrated with 0.1 N NaOH in the presence of phenolphthalein and acidity value was determined with the following equation:

(2) $$A=\frac{282\times N\times 100\times V}{1000\times W}\times 100$$

where *N* indicates normality of sodium hydroxide (NaOH), *V* presents the volume of consumed NaOH, *W* represents the weight of sample (g), and *A* expresses fatty acids content based on oleic acid in 100 g sample.

Peroxide value (PV) was analyzed according to AOCS Cd 8‐53, 1993. Briefly, 5 g oil was moved into a beaker (250 ml), then 300 ml acetic‐acid–chloroform with the proportion of 2:3 was added; after homogenization, 0.5 ml saturated iodide potassium was added and was left for 1 min in darkness. In the final solution, 30 ml distilled water was added and followed by titration using sodium thiosulfate 0.1 M till obtaining a yellow color. PV was determined as:

(3) $$P=\frac{S\times M\times 100}{W}$$

where *S* is the consumed volume of sodium thiosulfate in mL, *M* indicates molarity of sodium thiosulfate, *W* represents the weight of sample (extracted oil) in g, and *P* demonstrates the PV based on milli equivalents of oxygen/kg of oil (meq. kg^−1^ oil).

### 2.3 Determination of total phenolic content

Total phenolic content (TPC) content was measured with Folin–Ciocalteu reagent. In this regard, 1 g of each sample was mixed with 3 ml methanol: water (90:10), homogenized for 4 min and centrifuged (1,008 *g*) for 5 min; then, 20 µl of the supernatant solution was mixed with 8.2 ml water and 0.5 ml Folin–Ciocalteu reagent. After 5 min, 1 ml sodium carbonate 10% was added to the solution and was left at darkness. After 1 hr, the absorbance was recorded using a spectrophotometer at 765 nm. To construct standard graph, gallic acid (0–1000 µg mL^−1^) was used, and the final TPC content was reported as mg GAE kg^−1^ Sample (Bail, Stuebiger, Krist, Unterweger, & Buchbauer, 2008).

### 2.4 Analysis of fatty acids profile by gas chromatography

Firstly, methyl esters of fatty acids were prepared and the analysis of fatty acid profile was done by AOCS Ce 2‐66, 1993. For this purpose, a gas chromatography (GC) equipped with silicon hairy column number 70 (length of 60 m and diameter of 0.25 µm) was used. The initial temperature was set on 80°C, and with a temperature enhancement rate of 15°C min^−1^, the temperature reached to 200°C which was kept for 10 min; then the temperature increased up to 220°C and was kept for the next 5 min. The temperature of injection valve and detector temperature was set on 210°C and the flow rate of gas (helium) was adjusted on 1 ml min^−1^. Finally, the obtained peak area by the GC was compared to the standard graph and the quantity of each fatty acid was detected and reported as percentage.

### 2.5 Determination of tocopherols by high‐performance liquid chromatography

Determination of tocopherols was accomplished according to the method of AOCS Ce 8‐89, 1993 with the use of high‐performance liquid chromatography (HPLC). In this regard, a column SI 60‐5 (LiChrosorb) with the diameter of 250 × 4.5 ml and particle size of 5 µm was applied along with a florescence indicator. Mobile phase of acetonitrile in combination of distilled water with the proportion of 95‐5 was selected and its rate was adjusted on 0.6 µl min^−1^. Based on the retention time of tocopherols and the obtained chromatogram of the injected oil samples, amount of tocopherols was determined.

### 2.6 Statistical analysis

Response surface methodology, with the use of Box–Behnken design was applied for the evaluation of the influence of independent variables including microwave irradiation power (*X*_1_), PEF intensity (*X*_2_), and press rotational speed (*X*_3_) on different responses. To evaluate response surface behavior, a multivariate second‐order equation was designed for each independent variable. Finally, to compare the fatty acid profiles and tocopherol levels, a factorial design by SAS software was applied. The comparison of obtained means was done by the Duncan test.

## 3 RESULTS AND DISCUSSION

### 3.1 The effect of microwave‐PEF pretreatments on oil extraction efficiency

To improve oil extraction efficiency from Niger seeds, second‐order multivariate model was applied as the optimized model (Table 1). Based on the ANOVA results as presented in Table 2, the linear effects of microwave time, PEF intensity and press speed on oil extraction efficiency were detected significant (*p* < .05). The effects of second‐order parameters of the studied variables on extraction efficiency were also significant, but the interactive effects between microwave time with PEF intensity were nonsignificant (*p* > .05). Also, it was found that screw press rotational speed and quadratic effect of microwave irradiation time had the highest effects on oil extraction efficiency.

**Table 1:** Model selection for dependent (response) variables

Models	Extraction efficiency		Refractive index		Chlorophyll		Acidity		Peroxide value		Phenolic compounds	
	Sum of squares	Pb > *F*	Sum of squares	Pb > *F*	Sum of squares	Pb > *F*	Sum of squares	Pb > *F*	Sum of squares	Pb > *F*	Sum of squares	Pb > *F*
Mean	12975.88		**37.14**		2.44		86.03		2990.40		2542000	
Linear	694.44	0.0098	0.000	1.0000	0.49	0.0015	2.85	0.0019	31.13	<0.0001	344100	0.0076
2FI	36.48	0.8592	0.000	1.0000	0.077	0.2375	0.18	0.6930	0.091	0.9742	47423.80	0.5138
Quadratic	**467.03**	**<0.0001**	0.000	1.0000	**0.15**	**<0.0001**	**1.23**	**<0.0001**	**3.73**	**0.0018**	175600	0.0006
Cubic	18.02	0.0015	0.000	1.0000	0.007816	<0.0001	0.0001	0.8494	0.55	0.0021	17894.26	0.0002
Residue	0.53		0.000		0.00005080		0.0005		0.019		163.43	
Total	14,192.37		37.14		3.17		90.29		3025.91		3127000	

**Table 2:** Analysis of variance (ANOVA) for determined parameters in oil extraction by microwave‐PEF pretreatment

Source	Extraction efficiency			Refractive index			Chlorophyll			Acidity			Peroxide value			Phenolic compounds		
	Sum of squares	*F* Value	Pb > *F*	Sum of squares	*F* Value	Pb > *F*	Sum of squares	*F* Value	Pb > *F*	Sum of squares	*F* Value	Pb > *F*	Sum of squares	*F* Value	Pb > *F*	Sum of squares	*F* Value	Pb > *F*
Model	1197.95	50.25	<0.0001	0.000			0.72	70.80	<0.0001	4.26	5459.73	<0.0001	34.95	48.11	<0.0001	567,100	24.43	0.0002
*X*_1_	19.85	7.49	0.0290	0.000	63,660,000	<0.0001	0.038	33.40	0.0007	0.34	3875.82	<0.0001	10.24	126.85	<0.0001	264,700	102.62	<0.0001
*X*_2_	120.98	45.65	0.0003	0.000	63,660,000	<0.0001	0.092	81.50	<0.0001	0.41	4,773	<0.0001	19.25	238.53	<0.0001	50505.57	19.58	0.0031
*C*_3_	553.61	208.98	<0.0001	0.000			0.36	324.48	<0.0001	2.10	24233.9	<0.0001	1.64	20.30	0.0028	28825.93	11.17	0.0124
*X*_1_ *X*_2_	0.040	0.015	0.9057				0.001156	1.03	0.3442	0.048	557.97	<0.0001	0.062	0.77	0.4080	42292.95	16.39	0.0049
*X*_1_ *X*_3_	10.89	4.11	0.0822				0.041	36.67	0.0005	0.0049	56.49	0.0001	0.013	0.16	0.6977	122.46	0.047	0.8337
*X*_2_ *X*_3_	25.55	9.65	0.0172				0.034	30.45	0.0009	0.13	1494.07	<0.0001	0.016	0.19	0.6732	5008.39	1.94	0.2062
*X*_1_^2^	234.59	88.56	<0.0001				0.010	9.22	0.0190	0.38	24233.9	<0.0001	0.60	7.41	0.0296	157,600	61.09	0.0001
*X*_2_^2^	109.16	41.21	0.0004				0.048	74.59	<0.0001	0.060	695.5	<0.0001	2.91	36.11	0.0005	8806.97	3.41	0.1071
*X*_3_^2^	77.55	29.28	0.0010				0.039	34.60	0.0006	0.76	8779.98	<0.0001	0.015	0.18	0.6803	1782.34	0.69	0.4333
Residual	18.54			0.000			0.007867			0.44			0.56			18057.69		
Pure Error	0.53			0.000			0.00005080			0.00624			0.019			163.43		
Cor Total	1216.49			0.000			0.72			4.26			35.51			585,200		

Abbreviation: PEF, pulsed electric fields.

**Figure 1:**
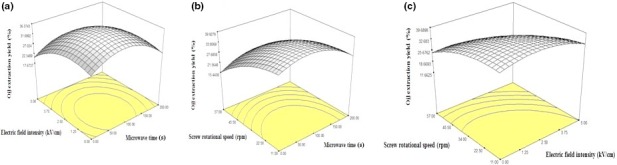
3D graphs of (a) microwave irradiation time and pulsed electric field intensity (b) microwave time and screw press rotational speed (c) pulsed electric field intensity and screw press rotational time on oil extraction efficiency

As shown in Figure 1, an increase in microwave irradiation time as well as PEF intensity firstly enhanced the oil extraction efficiency but with more enhancements of the above‐mentioned parameters, extraction efficiency was decreased. Improvement in oil extraction efficiency by microwave irradiation could be related to creation of more fracture in the cells containing oil during microwave pretreatment (Bakhshabadi, Mirzaei, Ghodsvali, Jafari, Ziaiifar, et al., 2017; Uquiche, Jeréz, & Ortíz, 2008). Moreover, it has been reported that increase of oil extraction efficiency when applying microwave may be due to the destruction/denaturation of proteins (Mohamed & Awatif, 1998). The achieved results of this section were in agreement with other studies (Momeny, Rahmati, & Ramli, 2012; Nde, Boldor, & Astete, 2015; Terigar, Balasubramanian, Sabliov, Lima, & Boldor, 2011). In terms of PEF, the causes of oil extraction efficiency enhancements may be attributed to electrical destruction of the cells and more penetration possibility of solvent into them (Schroeder, Buckow, & Knoerzer, 2009) which is similar to the results of other researchers (Bakhshabadi et al., 2018; Guderjan, Töpfl, Angersbach, & Knorr, 2005). Further decrease in oil extraction efficiency at higher microwave irradiation time and PEF intensity could be explained by more destruction of internal structures of cells and blocking of oil extraction pores/routes. Bakhshabadi et al. (2018) demonstrated that application of high PEF intensity may lead to a decrease in oil extraction efficiency which were in agreement with the obtained results of the current study. Increase in screw press rotational speed led to a decrease in oil extraction efficiency possibly due to a reduction in applied pressure onto the seeds which is similar to the data reported by some other researcher groups (Deli, Farah Masturah, Tajul Aris, & Wan Nadiah, 2011; Evon, Vandenbossche, Pontalier, & Rigal, 2007). Table 3 (model 1) presents the finalized and optimized models of different responses for extraction efficiency of oil from Niger seeds.

**Table 3:** Designed equation models for dependent variables

Number	Dependent variables	Equations	*R* ^2^	*R* ^2^‐adj	CV
1	Extraction yield	Y = +35.56 − 1.58 *X*_1_ − 3.89 *X*_2_ − 8.32 *X*_3_ + 0.10 *X*_1_ *X*_2_ + 1.65 *X*_1_ *X*_3_ − 2.53 *X*_2_ *X*_3_ − 7.46 *X*_1_^2^ − 5.09 *X*_2_^2^ − 4.29 *X*_3_^2^	0.9848	0.9652	5.89
2	Refractive index	Y = +1.48	0.00	0.00	0.00
3	Chlorophyll	Y = +0.51 + 0.069 *X*_1_ + 0.11 *X*_2_ + 0.21 *X*_3_ + 0.017 *X*_1_ *X*_2_ + 0.10 *X*_1_ *X*_3_ + 0.093 *X*_2_ *X*_3_ −0.050 *X*_1_^2^ − 0.14 *X*_2_^2^ − 0.096 *X*_3_^2^	0.9891	0.9752	8.84
4	Acidity (%)	Y = +1.96 + 0.21 *X*_1_ + 0.23 *X*_2_ + 0.51 *X*_3_ − 0.11*X*_1_ *X*_2_ − 0.035 *X*_1_ *X*_3_ + 0.18 *X*_2_ *X*_3_ + 0.30*X*_1_^2^ − 0.12 *X*_2_^2^ + 0.43*X*_3_^2^	0.999	0.997	0.41
5	Peroxide value	Y = +12.67 + 1.13 *X*_1_ + 1.55 *X*_2_ + 0.45 *X*_3_ + 0.12 *X*_1_ *X*_2_ − 0.058 *X*_1_ *X*_3_ − 0.063 *X*_2_ *X*_3_ + 0.38 *X*_1_^2^ + 0.83 *X*_2_^2^ + 0.060 *X*_3_^2^	0.9841	0.9636	2.14
6	Phenolic compounds	Y = +264.47 + 181.91 *X*_1_ + 79.46 *X*_2_ + 60.03 *X*_3_ − 102.83 *X*_1_ *X*_2_ + 5.53 *X*_1_ *X*_3_ + 35.38 *X*_2_ *X*_3_ + 193.46*X*_1_^2^ + 45.73 *X*_2_^2^ + 20.57 *X*_3_^2^	0.9691	0.9295	13.13

### 3.2 The effects of independent variables on the quality of extracted oil

According to the results in Table 1, all evaluated models on the refractive index (RI) of extracted oil were not significant (*p* > .05) and the effects of all variables including microwave time, PEF intensity, and screw press rotational speed on RI were almost not significant. The value of RI for all of the studied samples was equal to 1.478. RI is mostly used as a criterion of the oil purity determination. This parameter increases at higher chain lengths of fatty acids (while the relation is not linear). Also, RI has considerable impacts on controlling the catalytic hydrogenation and isomerization processes. Moreover, temperature and saturation rate are effective on RI as well (Uquiche et al., 2008). The findings of the present study were in agreement with the obtained results by Tale Masouleh, Asadollahi, and Eshaghi (2015).

**Figure 2:**
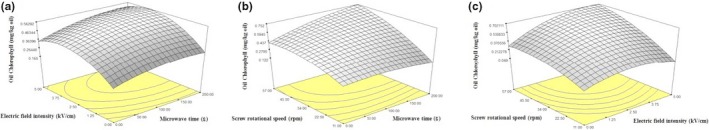
3D graphs of (a) microwave irradiation time and pulsed electric field intensity (b) microwave time and screw press rotational speed (c) pulsed electric field intensity and screw press rotational speed on chlorophyll content of the extracted oil

The ANOVA results in Table 2 revealed that the linear effect of microwave irradiation time, PEF intensity and screw press rotational speed on chlorophyll content of the extracted oil samples was significant in the confidence level of 0.001. The quadratic and interactive effects of the studied parameters (with an exception in interactive effect between microwave time and PEF intensity) were also detected significant on chlorophyll content of the extracted oil. Based on the results of Table 1, the most optimized model explaining the effects of microwave irradiation on chlorophyll content was Quadratic. Figure 2 indicates the interactive effects of microwave irradiation time and PEF intensity on chlorophyll content of extracted oils from Niger seeds. It can be seen that at higher rates of microwave irradiation time and PEF intensity, chlorophyll content increases, which is more obvious in low irradiation times and PEF intensities. Moreover, with the increase in screw press rotational speed, chlorophyll content improves. The possible reason of chlorophyll increase with enhancements in microwave time, PEF intensity, and screw press speed may be the high solubility and penetration of the chlorophylls into the extracted oil sample as reported in the study of (Megahed, 2001).

From the *F* value and the fitted model for chlorophyll content, it may be explained that screw press rotational speed had the most considerable effect on the chlorophyll extraction. Chlorophylls are tetra pyrolytic pigments obtained from green leaves with a high impact on the maintenance of plants via engaging in photosynthetic reactions. Chlorophylls with the absorbance of light energy transmit the stored energy into appropriate energy transmitters in order to be used in carbohydrate synthesis via water and carbonic gas usage. Unlike carotenoids, chlorophylls and particularly chlorophyll a is so sensitive against light. This type of chlorophyll after energy absorbance may transmit energy into triple oxygen and produce high‐activated form of singlet oxygen; as a result, it might react with β‐carotene and unsaturated fatty acids (Gómez‐Alonso, Mancebo‐Campos, Salvador, & Fregapane, 2007). Higher chlorophyll content may increase the color indices; in other words, color enhancements of the extracted oils may be attributed to rupture of plant tissues during treatment and thus lead to increase in pigment extraction efficiency. In this regard, the effects of different temperatures during roasting of safflower seeds on color deterioration of the extracted oil was studied by Lee, Oh, Chang, and Kim (2004). They demonstrated that color creation in oil was affected by the roasting temperatures, and at higher temperature, the color of extracted oil was changed from yellow to dark brown. It has also been reported that the application of PEF may lead to enhancements of color in the extracted oil (Bakhshabadi, Mirzaei, Ghodsvali, Jafari, Ziaiefar, et al., 2017; Guderjan, Elez‐Martínez, & Knorr, 2007).

**Figure 3:**
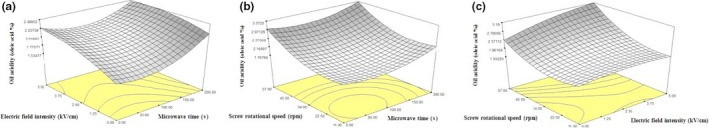
3D graphs of (a) microwave irradiation time and pulsed electric field intensity (b) irradiation time and screw press rotational time (c) pulsed electric field intensity and screw press rotational speed on oil acidity

The provided data in Table 2 as well as suggested model for the acidity value of the extracted oil in Table 3 revealed that the highest effect on acidity value for the extracted oil was in conditions when the combined pretreatment of microwave‐PEF intensity was applied which affected the oil extraction significantly by linear and quadratic modes of screw press rotational speed. On the other hand, it was observed that an increase in microwave time, PEF intensity and press rotational speed may lead to an enhancement in acidity value of the extracted oil (Figure 3). This could be attributed to higher activity of lipase enzyme leading to free fatty acid production which are considered as undesirable compounds in edible oils (Guderjan et al., 2007). Puértolas and de Marañón (2015), reported similar results to the present study. At higher press speeds, the oil acidity enhancement occurs which might be due to temperature increase during heating process (Puértolas & de Marañón, 2015) which is in agreement with previous studies (Amalia Kartika, Pontalier, & Rigal, 2005; Sriti et al., 2012). Finally, oil acidity increase at higher microwave irradiation times might be attributed to the chemical degradation of triglycerides and production of free fatty acids. Lipolytic enzymes like lipase are placed in the internal parts of cells; in normal cells, these enzymes are not able to attack fats/oils. But during extraction and at high temperatures, the membranes of cells are damaged physically, therefore lipase enzyme starts its activities (Ghavami, Gharachorloo, & Ezatpanah, 2003). The achieved results of this section were in agreement with the study of Kittiphoom and Sutasinee (2015) and Veldsink et al. (1999); but, opposite to the results of Uquiche et al. (2008) (Kittiphoom & Sutasinee, 2015; Uquiche et al., 2008; Veldsink et al., 1999).

**Figure 4:**
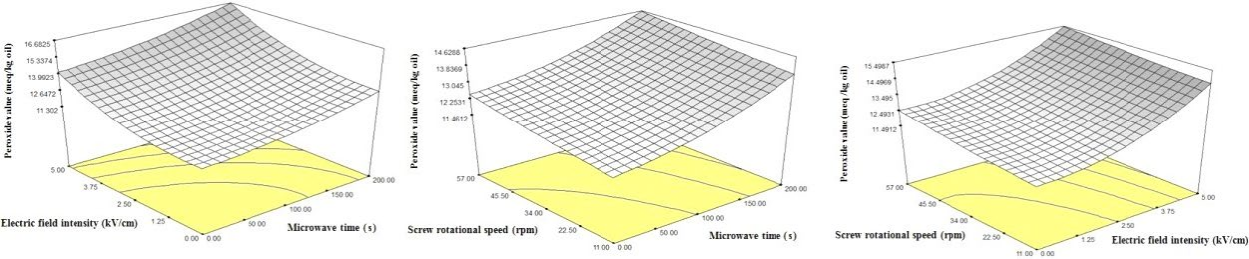
The effects of independent variables on total phenolic compounds of oils

Peroxide value is associated with hydroperoxides within extracted oil which is affected by temperature, time, and fatty acid profile (Zhang et al., 2010). Higher PVs show a developed production of secondary products by lipid oxidation such as carbonyls, aldehydes and conjugated di‐en; therefore, PV determination is an essential factor for oxidation process analysis. Our results in Table 2 revealed that the linear effects of microwave time, PEF intensity, and screw speed on the PV were significant (*p* < .001). Moreover, the quadratic effects (except with screw press rotational speed) unlike the interactive effects were also significant. As shown in Figure 4, with an increase in PEF intensity, PV of the extracted oils increased which may be attributed to the high oxidation of fatty acids at higher temperatures when PEF intensity increased (Guderjan et al., 2007). Zeng et al., (2010) reported similar results. Also, higher microwave irradiation times, due to temperature enhancements, might lead to a higher oxidation rate and then, increased PVs; these results were in agreement with the some other studies (Hassanein, El‐Shami, & El‐Mallah, 2003; Valentova, Novotna, Svoboda, Schwarz, & Kas, 2000). Finally, an increase in screw press rotational speed, resulted in higher PVs of the extracted oils. Table 3 presents the obtained models of PV for the oils extracted by combined PEF‐microwave pretreatment demonstrating a high impact of PEF intensities on the PVs.

**Figure 5:**
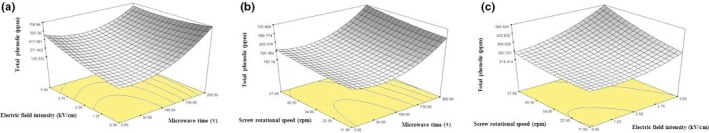
3D graphs of (a) microwave irradiation time and pulse electric field (b) microwave irradiation time and screw press rotational time (c) pulsed electric field intensity and screw rotational speed on total phenolic compounds content

Phenolic compounds are an important group of secondary metabolites with antioxidant activities due to having hydroxyl groups in their structures. The application of phenolic compounds in food industry is increasing, because these compounds may retard lipid oxidation and improve qualitative and nutrition properties of food products (Assadpour, Jafari, & Esfanjani, 2017; Faridi Esfanjani, Assadpour, & Jafari, 2018). According to ANOVA results, the linear effects of different parameters on phenolic compounds were significant. As shown in Figure 5, at higher microwave irradiation times, PEF intensity, and screw speeds, total phenolic compounds of the extracted oils were increased. The heating effect of microwave energy is associated with its direct effects on molecules with dipolar rotational mechanisms and ionic transmission. Polar molecules such as phenolic compounds and ionic solutions absorb microwave energy due to having dipolar torque leading to temperature enhancements and increase of their transfer into the extracted oils (Proestos & Komaitis, 2008) as shown in some other studies as well (Beejmohun et al., 2007; Jiao et al., 2014). Also, Rombaut et al. (2015) reported that at higher press rotational speeds, total phenolic compounds increased.

Table 3 indicates that fitted models for each response in the studied conditions have exact and accurate capability of fitting experimental data. The achieved correlation coefficients and the adjusted correlation coefficients as well as root mean squared errors (RMSE) indicate the appropriateness and fitness of the selected models in the present study (Table 3).

### 3.3 Optimization of oil extraction process from Niger seeds

To find the optimized conditions of oil extraction from Niger seeds with the use of microwave‐PEF in the range of applied independent variables, (microwave time of 0–200 s, PEF intensity at 0–5 kV/cm and press rotational speed ranged from 11–57 rpm), the targets were considered as the maximum oil extraction efficiency and total phenolic compounds content, and the minimum acidity and PVs. The desirability in the optimized conditions was determined as 0.789. It was found that microwave time of 156.34 s, PEF intensity of 1.18 kV/cm, and press speed of 20 rpm were the best conditions so that an oil with 35% extraction efficiency, RI = 1.478, acidity of 1.94%, and total phenolics of 410 ppm will be produced.

### 3.4 Influence of microwave‐PEF pretreatment on the fatty acid profile of oil samples

**Figure 6:**
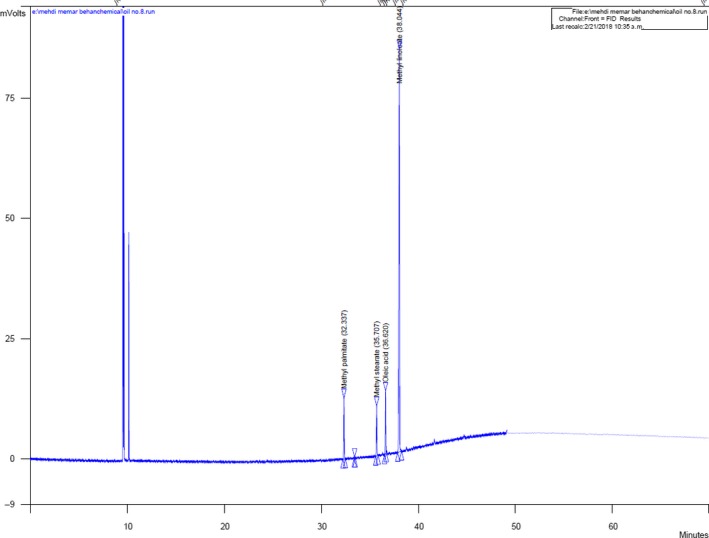
A typical GC‐MS chromatogram for determination of fatty acid profile in Niger seed oil. GC, gas chromatography

The profile of fatty acids in extracted oil of Niger seeds both at microwave‐PEF conditions and standard sample has been shown in Table 4 and Figure 6. In both samples, linoleic acid was the most predominant fatty acid in the oil of Niger seeds. As mentioned, when combined microwave‐PEF is used as a pretreatment in oil extraction process, the quantity of unsaturated fatty acids (oleic and linoleic acids) decreased and the amount of saturated fatty acids (palmitic and stearic acids) increased (*p* < .05). This might be attributed to the susceptibility of unsaturated fatty acids against high temperatures. It has been reported previously that the predominant fatty acid in Niger seeds is linoleic acid which may be varied based on the variety of seeds (Getinet & Teklewold, 1995; Pradhan et al., 1995). Also, the composition of fatty acids in oilseeds might be affected by variety, cultivation conditions, and ripening grade of plants up to their processing (Murkovic, Hillebrand, Draxl, Pfannhauser, & Winkler, 1997). The relevant effects of different pretreatments on fatty acid profile may be attributed to the slight alterations in fatty acids. For instance, our results in this section were in agreement with those obtained by Murkovic et al. (1997) who demonstrated that stearic acid is one of the most stable detected fatty acids and is not changed under different conditions. (Kim et al., 2002) also reported that application of microwave in oil extraction did not have considerable effects on fatty acid profile; on the other hand, Ariza‐Ortega, Ramírez‐Moreno, Ramos‐Cassellis, and Díaz‐Reyes (2014) revealed that by applying PEF, some alterations on unsaturated fatty acids is observed. The achieved results by Zeng et al. (2010) showed that application of PEF pretreatment in oil extraction of peanut increased stearic and palmitic acids contents but decreased oleic and linoleic acids, that were in agreement with the findings of the present study.

**Table 4:** Fatty acids composition of the extracted oil with the use of optimize treatment

Fatty acid	Molecular formulation	Standard	Optimized oil
		Fatty acid content (%)	
Palmitic acid	C16	8.34 ± 0.05^bC^	8.76 ± 0.01^aB^
Stearic acid	C18	8.09 ± 0.01^aD^	8.15 ± 0.01^aC^
Oleic acid (ω‐9cis)	C18:1(9)	8.90 ± 0.02^aB^	8.77 ± 0.05^aB^
Linoleic acid	C18:2(9,12)	74.64 ± 0.03^aA^	74.32 ± 0.01^bA^

Note

The similar capital and small letter in each column and row respectively demonstrate nonsignificant difference in confidence level of 0.05.

### 3.5 The effects of microwave‐PEF pretreatment on tocopherol content of the extracted oil

**Figure 7:**
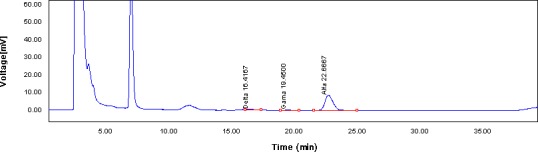
A typical HPLC chromatogram for determination of tocopherols in Niger seed oil. HPLC, high‐performance liquid chromatography

The comparison of tocopherol content determined in extracted oil samples revealed that α‐tocopherol was the most predominant tocopherol in both the optimized and standard samples followed by γ‐ and Δ‐tocopherols (Table 5 and Figure 7). As observed, application of combined microwave‐PEF pretreatment may lead to 2.79% increase in tocopherol content of extracted oils from Niger seeds, particularly in α‐ and Δ‐tocopherols which could be explained in lower reactions between these natural antioxidants and polysaccharides, proteins and different peptides within the seeds (Hamid Bakhshabadi, Mirzaei, Ghodsvali, Jafari, Ziaiifar, et al., 2017). Our results were in agreement with the obtained data of Wiktor et al. (2015) and Oomah, Liang, Godfrey, and Mazza (1998) who reported enhancements of vitamin E in grape oil extracted by the assistance of microwave pretreatment; the highest amount of tocopherols were determined during treatment time of 9 min. It was also reported an increase in tocopherol compounds in oil with the use of microwave pretreatment (Azadmard‐Damirchi et al., 2010; Moreau, Hicks, & Powell, 1999). Tocopherols are important natural antioxidants and by trapping intermediate hydroxides lead to a delay or retardation of oxidation chain; moreover, α‐tocopherols have health‐promoting and nutritional activity for humans (Silva, Pinto, Carrola, & Paiva‐Martins, 2010).

**Table 5:** Tocopherols of Niger seed oil achieved by optimized pretreatment

Tocopherols type	Standard	Optimized sample
α‐Tocopherols (%)	478.68 ± 7.5^aA^	488.62 ± 2.5^aA^
Δ‐Tocopherols(%)	9.14 ± 2.6^bC^	18.17 ± 0.2^aB^
γ‐Tocopherols (%)	27.80 ± 2.2^aB^	23.85 ± 0.3^aB^
Total tocopherols	515.62 ± 11.3	530.02 ± 3.0

Note

The similar capital and small letter in each column and row respectively demonstrating non‐significant difference in confidence level of 0.05.

## 4 CONCLUSION

The results of present study demonstrated that with an increase in microwave irradiation time, PEF intensity and screw press rotational speed, chlorophylls content, acidity and PVs, and total phenolic compounds of the extracted oils from Niger seeds were increased, while extraction efficiency showed an enhancement at the beginning and then decreased. The RI of extracted oil was constant at 1.478 and was not affected by pretreatments. The optimized extraction conditions were microwave irradiation time of 156.34 s, PEF intensity of 1.18 kV/cm, and screw press rotational speed of 20 rpm. The characterization of fatty acid profile of the extracted Niger seed oil presented that linoleic acid was the predominant fatty acid, and the application of microwave‐PEF pretreatment may lead to a decrease in unsaturated fatty acids and enhancements of saturated fatty acids content. The results of HPLC analysis showed that α‐tocopherols had the highest quantity among the tocopherols in Niger seed oil and applying combined microwave‐PEF pretreatment may lead to an increase in tocopherol content of the extracted oil. At the end, it may be suggested that application of microwave‐PEF pretreatment might be useful as an appropriate pretreatment in oil extraction industries.

## CONFLICT OF INTEREST

All authors declare that there is no conflict of interest.

## ETHICAL APPROVAL

There was no human or animal testing in this study.
